# The genetics of feed conversion efficiency traits in a commercial broiler line

**DOI:** 10.1038/srep16387

**Published:** 2015-11-10

**Authors:** Henry Reyer, Rachel Hawken, Eduard Murani, Siriluck Ponsuksili, Klaus Wimmers

**Affiliations:** 1 Leibniz Institute for Farm Animal Biology, Institute for Genome Biology, Wilhelm-Stahl-Allee 2, 18196 Dummerstorf, Germany; 2 Cobb-Vantress Inc., Siloam Springs, AR 72761-1030, United States of America; 3 Leibniz Institute for Farm Animal Biology, Research Group Functional Genome Analysis, Wilhelm-Stahl-Allee 2, 18196 Dummerstorf, Germany

## Abstract

Individual feed conversion efficiency (FCE) is a major trait that influences the usage of energy resources and the ecological footprint of livestock production. The underlying biological processes of FCE are complex and are influenced by factors as diverse as climate, feed properties, gut microbiota, and individual genetic predisposition. To gain an insight to the genetic relationships with FCE traits and to contribute to the improvement of FCE in commercial chicken lines, a genome-wide association study was conducted using a commercial broiler population (n = 859) tested for FCE and weight traits during the finisher period from 39 to 46 days of age. Both single-marker (generalized linear model) and multi-marker (Bayesian approach) analyses were applied to the dataset to detect genes associated with the variability in FCE. The separate analyses revealed 22 quantitative trait loci (QTL) regions on 13 different chromosomes; the integration of both approaches resulted in 7 overlapping QTL regions. The analyses pointed to acylglycerol kinase (*AGK*) and general transcription factor 2-I (*GTF2I*) as positional and functional candidate genes. Non-synonymous polymorphisms of both candidate genes revealed evidence for a functional importance of these genes by influencing different biological aspects of FCE.

The sustainable and efficient usage of resources in animal production is a major concern in agriculture. Over the last decade, increasing amounts of agricultural land and plant products have been used for ethanol and energy production, raising the economic pressure on animal production through higher feed costs^1^. Studies of livestock species showed that including feed efficiency as a trait in breeding schemes provides the potential to save feeding costs and resources while increasing productivity and reducing greenhouse gas emission (see, e.g.,^2^,^3^,^4^). In accordance with most performance-related traits, current efforts to increase feed efficiency in broilers are primarily related to genetic selection (about 85–90%); feeding strategies and management account for approximately 10–15% of phenotypic improvement^5^.

The most widely used measurement of individual feed conversion efficiency is the feed conversion ratio (FCR = intake/weight gain), due to its standardized on farm recording with appropriate feeder systems and the simple calculation. Feed conversion ratio accounts for the feed intake necessary to gain in body weight over a defined time period and results in an animal-specific value indicating better (low FCR) or worse (high FCR) efficiency. The complex biology of FCR is affected by processes interrelating feed intake and energy homeostasis. At the molecular level, signaling molecules within the bilateral gut-brain axis contribute to the regulation of feed intake. In this context, the efficiency of enteral absorption processes influences the availability of nutrients, thereby involving factors such as leptin, ghrelin, neuropeptide Y, and cholecystokinin (reviewed by^6^). Downstream of nutrient uptake, the efficiency of energy production (e.g., oxidative phosphorylation) contributes to cellular energy levels. Moreover, weight gain as a measurable component of feed efficiency is influenced by individual differences in energy requirement for growth, maintenance, and thermogenesis. Thus, there exists the potential for a variety of genes to affect FCR.

Quantitative trait loci (QTL) linked to feed intake and weight gain, deposited in the chicken QTL database, offer insight to the general genetic relationships and biological background of feed efficiency traits (http://www.animalgenome.org/cgi-bin/QTLdb/GG/index)^7^. However, the majority of known QTL regions were identified by microsatellite analysis and established in extreme experimental crosses, revealing large genomic regions that are suggested to explain differences in divergent lines rather than phenotypic variance in a terminal commercial line^8^. This highlights the necessity of verifying the segregation of these QTL in established commercial lines and obtaining genetic markers and candidate genes useable for implementation in commercial breeding schemes and marker-assisted selection^7^,^9^.

The current study was designed to investigate the genetics of FCE and weight traits in a commercial broiler population that is under selection for average daily gain and feed conversion over many generations. To achieve this aim, the Illumina chicken 60 K SNP chip, which provides moderate-density genotypes to increase the resolution and accuracy of QTL mapping^10^, was used. Included single nucleotide polymorphism (SNP) markers are distributed over the whole chicken genome and cover all 5 macro- and 5 intermediate chromosomes, 18 out of the 28 microchromosomes, both sex chromosomes, and two unmapped linkage groups. The utility of the 60 K SNP chip for genome-wide association studies (GWAS) was previously demonstrated in several studies of chicken diseases and performance-related traits (see, for example,^11^,^12^). Additionally, the results of single-marker analyses and a sophisticated Bayesian multi-marker approach were integrated to identify genomic regions and corresponding genes as an important step towards the development of new molecular markers for feed efficiency traits in poultry production.

## Results

### Population structure and linkage disequilibrium (LD)

**Figure 1:**
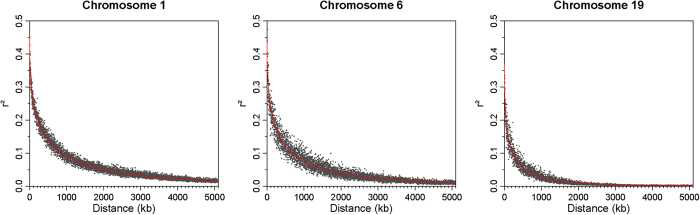
Values of LD versus physical distances on representations of macro- (chromosome 1), intermediate (chromosome 6), and microchromosomes (chromosome 19). Values of r^2^ between markers were averaged in non-overlapping windows of 1 kb and plotted against the distances between SNP markers. Nonlinear curves were fitted to estimate the average distance between markers showing extent of ‘useful’ LD (r^2^ ≥ 0.25).

Population stratification was not prominent in the analyzed Cobb broiler group (n = 862) as revealed by a multidimensional scaling plot of the first two dimensions of genetic distances between individuals (Supplementary Figure S1). Chromosome-wide linkage analysis was performed to evaluate the prediction accuracy of QTL mapping. Therefore, mapped SNP were used to investigate the density and the extent of LD of markers on micro-, intermediate, and macrochromosomes in the studied broiler population. The analyses revealed differences in the distance and linkage between adjacent markers on micro-, intermediate, and macrochromosomes (Fig. 1, Table 1). Neighboring markers on macrochromosomes have an average distance to each other of 29 kb and showed an extent of LD of r^2^ = 0.35. On these chromosomes, markers with distances smaller than 115 kb were predicted to have an extent of ‘useful’ LD (r^2^ ≥ 0.25). On intermediate chromosomes, markers mapped, on average, every 20 kb and showed a similar extent of LD (r^2^ = 0.34) compared to markers on macrochromosomes. Markers with genomic distances smaller than 85 kb showed an extent of ‘useful’ LD. The density of mapped markers on microchromosomes was higher, with about one marker every 13 kb, but the extent of LD between adjacent markers was only r^2^ = 0.29. Markers on microchromosomes showed an extent of LD with r^2^ ≥ 0.25 up to distances of approximately 20 kb.

### Body weight traits

**Figure 2:**
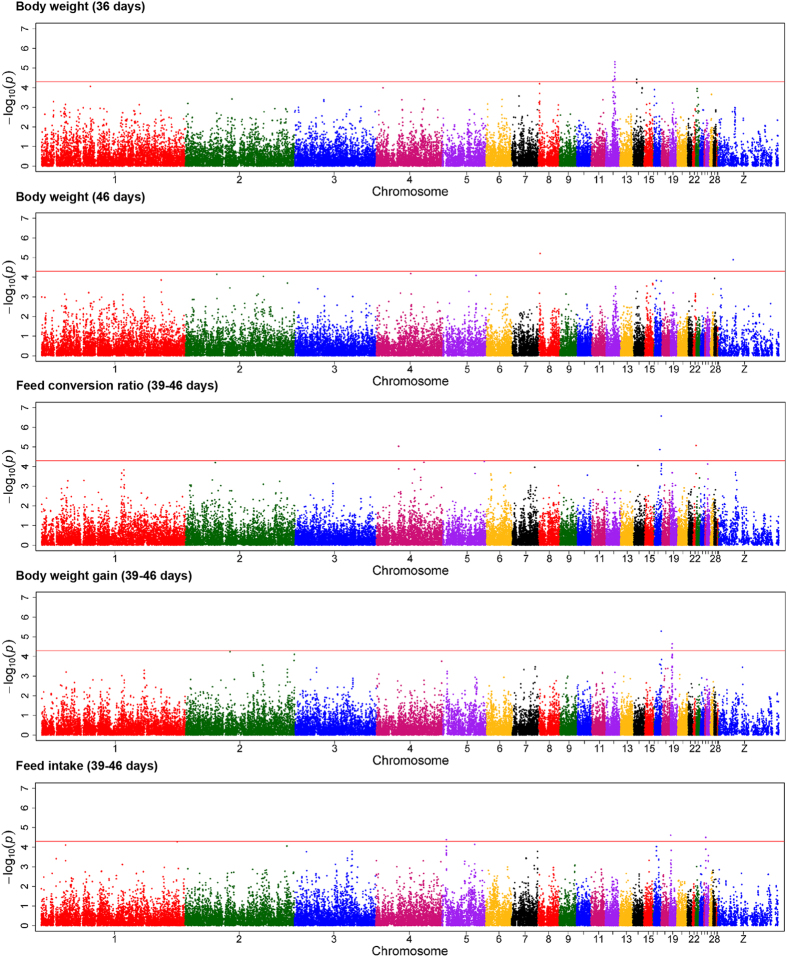
Manhattan plots of genome-wide association analysis results for body weight and feed efficiency traits in a commercial broiler line (n = 859) using single-marker analysis implemented in a generalized linear model. Chromosomes 29 and 30 represent linkage groups LGE22C19W28_E50C23 and LGE64, respectively. The threshold for suggestive significance was set at -log_10_(p-value) = 4.3. Detailed information about SNP exceeding the threshold levels are listed in Supplementary Table S2.

Markers were analyzed for segregation with body weights, recorded at the end of grower (BW36) and finisher (BW46) phases, by Bayesian GWAS. Consistent QTL regions were identified between 1.0 and 2.0 Mb on chromosome 8 and between 13.0 and 14.0 Mb on chromosome 12 (Table 2). The 1-Mb window on chromosome 8 explained 0.74 and 1.59% of the genetic variance of BW36 and BW46, respectively. Additionally, this region was further indicated by generalized linear model (GLM) analysis for BW46, and the most dominant SNP, rs16617885, mapped in an intergenic region between *PTPRC* and *NR5A2* (Fig. 2).

**Figure 3:**
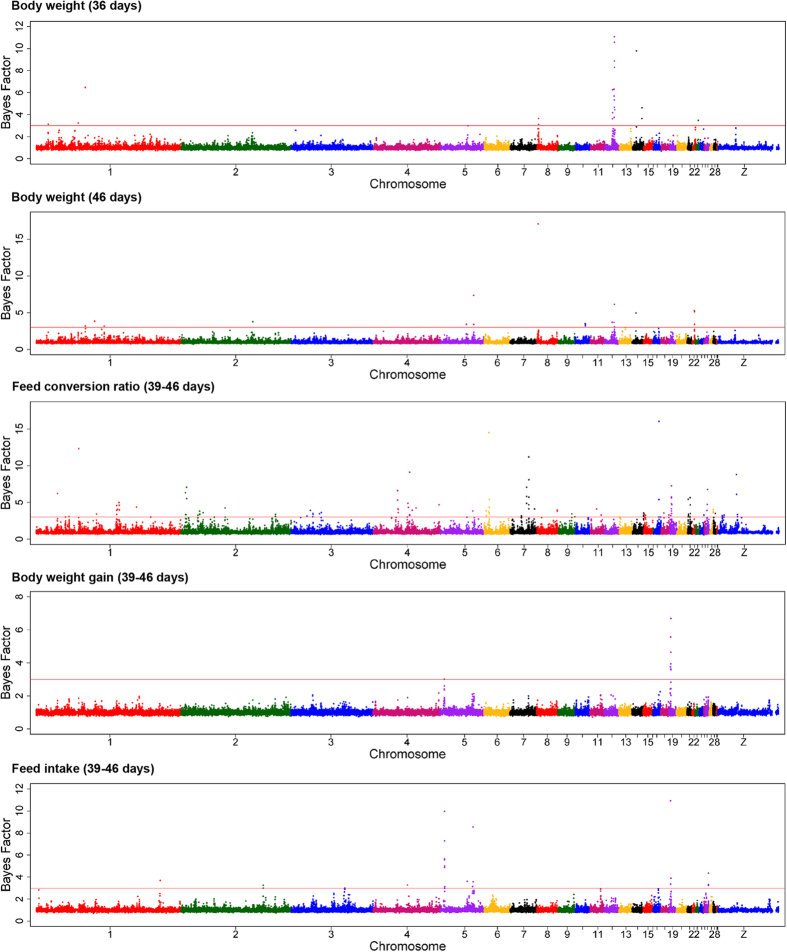
Manhattan plots for genome-wide association using a Bayesian multi-marker approach (Bayes B). Observed traits were body weight at 36 and 46 days of age and feed conversion ratio, body weight gain, and feed intake recorded during a feeding trial between days 39 and 46. The horizontal line represents the threshold of suggestive linkage with the trait at Bayes Factor = 3. Chromosome 29 represents linkage group LGE22C19W28_E50C23, and chromosome 30 is linkage group LGE64. Detailed information about SNP exceeding the threshold levels are listed in Supplementary Table S3.

The indicated QTL region between 13.0 and 14.0 Mb on chromosome 12 was supported by an adjacent region (12.0–13.0 Mb), which explained the highest proportion of the genetic variance of BW36 (5.64%). Within this region, 8 SNP reached the significance threshold (-log_10_[p-value] ≥ 4.3) in the GLM analysis and 9 SNP markers showed Bayesian factors (BF) above 3 (Figs 2 and 3). The window covers two large genes, *FHIT* and *PTPRG*, and the miRNA gaa-mir-1550. The highest associated SNP, rs13612706 (-log_10_[p-value] = 5.31; BF = 11.07), and 5 other top-ten SNP mapped within the 0.37-Mb region spanning the *PTPRG* gene.

The combination of GLM and Bayesian analyses using BW36 as the response variable revealed two other QTL regions, on chromosomes 12 and 14, each explaining 0.99% of the genetic variance (Table 2).

### Feed conversion efficiency traits

For FCR, the integration of Bayesian and GLM analyses revealed one overlapping QTL region on chromosome 17 (Table 2). The 1-Mb window between 7.0 and 8.0 Mb explained 0.74% of total genetic variance. The highest significantly associated marker in this window, rs14098962, mapped beside the *RXRA* gene in a region with a high gene density. Moreover, a second region was detected on chromosome 17, mapping between 8.9 and 9.9 Mb (Fig. 2). This window was derived by SNP GGaluGA117403, which was the only SNP in the GLM analyses exceeding the Bonferroni-corrected genome-wide significance threshold. The marker mapped to an intronic region of the *GPR144* gene. Five additional QTL regions for FCR, located on chromosomes 1, 4, 6, 7 and 19, were identified using Bayesian GWAS (Table 2). Among these, the highest proportion of the genetic variance of FCR (0.95%) was in a 1-Mb window between 6.0 and 7.0 Mb on chromosome 6. In this window, SNP rs14568465 showed high evidence for association with the trait in Bayesian GWAS (BF = 14.50; Fig. 3) and mapped in the *CTNNA3* gene.

**Figure 4:**
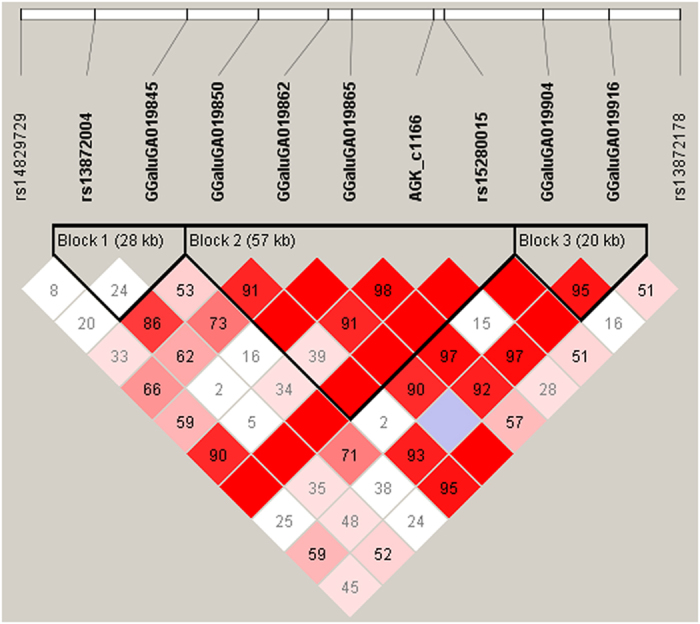
Linkage map of markers spanning the genomic region including the *AGK* locus on chicken chromosome 1. Depicted is the LD (D’ value in diamond) between markers including genotype information of 240 broilers also genotyped for *AGK* SNP c.1166. Linkage blocks were defined using the ‘solid spine of LD’ algorithm implemented in the Haploview 4.2 software.

Moreover, Bayesian GWAS revealed a QTL region between 57.0 and 58.0 Mb on chromosome 1. In this 1-Mb window, SNP GGaluGA019865, showing evidence (BF = 12.3; Fig. 3) for association with FCR, is located in the first intron of the acylglycerol kinase encoding gene (*AGK*). Linkage analysis between markers in this region showed that the SNP GGaluGA019865 is part of a 57-kb spanning linkage group that covers only *AGK* (Fig. 4). Information provided by the SNP database dbSNP was used to genotype the *AGK* SNP c.1166 G > A. The SNP is located in the coding region of the candidate gene and leads to a non-synonymous amino acid exchange (R389H), giving it high potential to be directly involved in phenotypic alterations^13^. The effects of the *AGK* substitution on the FCE traits and body weight are shown in Table 3. The alternative A allele, with significantly higher FCR values, showed a tendency to associate with lower BWG without effects on FI. Furthermore, the substitution had significant effects on body weight at 36 but not at 46 days of age.

**Figure 5:**
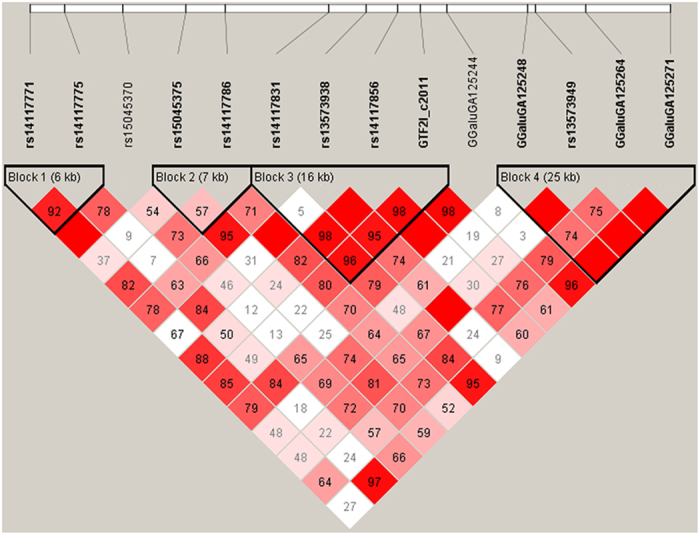
Linkage map of the genomic region including the *GTF2I* locus on chicken chromosome 19. Depicted is the LD (D’ value in diamond) between markers including genotype information of 240 broilers also genotyped for *GTF2I* SNP c.2011. Linkage blocks were defined using the ‘solid spine of LD’ algorithm implemented in the Haploview 4.2 software.

The GWAS for body weight gain (BWG) revealed two QTL regions on chromosomes 17 and 19, completely overlapping with regions also linked to FCR (Table 2). The QTL on chromosome 19 between 2.0 and 3.0 Mb explained 2.45% of the genetic variance of BWG and was further supported by two significantly associated markers obtained from GLM analysis (Fig. 2). The most prominent marker in this region mapped to an intronic region of the general transcription factor 2-I (*GTF2I*) gene. The aggregation of Bayesian and GLM analyses of feed intake (FI) provided evidence for QTL regions on chromosomes 5 and 19 (Table 2). The region on chromosome 5 between 5.0 and 7.0 Mb comprises two adjacent 1-Mb windows explaining 0.92 and 1.44% of the genetic variance of FI, respectively. The most prominent markers in both regions mapped in and next to the *SPON1* gene. The QTL region on chromosome 19 also comprised two adjacent 1-Mb windows spanning the region between 1.0 and 3.0 Mb. The 1-Mb window between 2.0 and 3.0 Mb completely overlaped with QTL regions for FCR and BWG. Out of the 8 genes that mapped in this section, the genes *GATSL2*, *WBSCR16*, *NCF1*, *GTF2I*, and *GTF2IRD1* are located next to the most prominent SNP rs14117856 obtained from single-marker GWAS of FCR and BWG. Analysis of the LD in this region supports *GTF2I* as the most plausible positional candidate gene (Fig. 5). Genotyping of *GTF2I* SNP c.2011 A > C – located in the coding sequence of *GTF2I* (p.K671Q) – showed significant association with BW46, FI, BWG, and FCR (Table 3). Carriers of the alternative allele showed higher BWG and FI during the feeding trial.

## Discussion

In total, the GWAS revealed 22 QTL regions on 13 different chromosomes indicated by either a contribution of a 1-Mb window to the genetic variance above the 0.5% in Bayesian approach, or the significant association (-log_10_[p-value] ≥ 4.3) of at least one SNP in the GLM analysis. The integration of both methods resulted in only 7 overlapping QTL regions. However, SNP with elevated Bayes factors in a QTL region also showed elevated -log_10_(p-values) in the single-marker approach. Irrespective of the threshold levels used in both GWAS approaches, the most prominent regions obtained showed a high overlap using both methods, arguing for a too-conservative significance threshold in the GLM analysis. Similarly, previous studies combining both methods for association analysis in livestock also revealed fewer QTL regions by GLM than by Bayesian GWAS^14^,^15^,^16^. In addition, it can be assumed that predominant variants with major effects on feed efficiency traits are largely fixed in a commercial population selected for feed efficiency over generations, and mainly those variants with low-to-moderate effect sizes contribute to phenotypic variation. The estimated heritability for FCR, FI, and BWG of meat-type chickens of the same age is moderate (0.41 to 0.48)^17^. This is not a contradiction, taking into account the multifactorial biological processes with contribution to the complex traits of feed efficiency.

The power of GWAS to detect variants with weak contributions is, in general, limited, but mainly depends on population size and is further affected by marker density and the extent of linkage disequilibrium between markers^16^,^18^. The linkage analysis of markers mapping on macro- and intermediate chromosomes revealed supportive evidence for genetic association, as depicted by approximately 4 adjacent markers. Microchromosomes – characterized by a high density of genes and high recombination rates^19^ – showed an average extent of ‘useful’ LD (r^2^ ≥ 0.25) that is slightly larger than the distances between adjacent markers. Therefore, the marker density, particularly on microchromosomes, is assumed to be insufficient to detect small segregating regions in the broiler population used in this and other studies^10^. Based on low effect size and the genetics of the traits, a Bayesian approach using 1-Mb windows seems to increase the prediction probability and shows overall better performance compared to single-marker analyses^14^,^20^. Furthermore, multi-marker analyses are suggested to show lower type I error rates compared to single marker regression approaches^21^. Nevertheless, the GLM approach – fitting only individual genotype classes in the model – revealed unique QTL regions not supported by multi-marker analysis. Thus, for example, the highest associated marker in the GLM approach, located on chromosome 17, showed no linkage in the Bayesian approach, but single-marker analysis revealed the heterozygous genotype as associated with effects on FCR and BWG. The homologous region harbors several candidate genes for weight- and growth-related traits previously shown in pigs^22^,^23^. One promising functional candidate within this region is sterogenic factor 1 (*NR5A1*), for which knock-out studies revealed elevated body weights related to late-onset obesity in mice^24^.

The consideration of body weight is highly important in poultry breeding and is thus a focus of many QTL studies. Not surprisingly, all 7 identified regions for body weight overlap with previously described large QTL regions obtained by using microsatellite-based analyses in crosses of divergent chicken lines (chicken QTL database,^7^). As indicated by our genome-wide and candidate gene association analyses, genetic factors affecting body weight traits are assumed to be highly age-dependent – e.g., depicted by differences between BW36 and BW46. Therefore, it is also likely that the genetics of FCE-related traits underlie age-specific differences, as previously discussed^17^.

The *PTPRG* gene demonstrated the strongest evidence for association with BW36 on chromosome 12. Interestingly, functional studies in mouse showed a significant association of *PTPRG* with body weight and bone mineral density^25^. In addition, genome-wide network analysis in the background of differential growth in cattle revealed the *FHIT* gene – located directly upstream of *PTPRG* – as one of the central hubs in the established growth network^26^. This provides an interesting candidate region for further analyses with focus on genetic differences affecting body weight in meat-type chicken lines. Among the QTL regions identified for FCE traits, two genes of interest were selected due to their positional and functional qualification. The *AGK* gene encodes a mitochondrial acylglycerol kinase, catalyzing the synthesis of phosphatidic and lyso-phosphatidic acids^27^. The products play roles as components of phospholipids and as signaling molecules, for instance in epidermal growth factor (EGF) signaling. Loss-of-function mutations of *AGK* in humans were shown to be causal for the occurrence of Sengers syndrome, suggestively based on dysfunction of mitochondrial lipid balance^28^. Thus, the observed effects of the *AGK* polymorphism on FCR in broilers are suggested to be related to mitochondrial function. Accordingly, laboratory analyses of patients affected by Sengers syndrome revealed shifts in the activity of respiratory chain complexes and citrate synthase, consequently effecting mitochondrial ATP synthesis^28^. Alterations in body growth and muscle structure related to Sengers syndrome are in accordance with observed effects on body weight and weight gain in broilers with divergent alleles of the *AGK* c.1166 G > A polymorphism.

The *GTF2I* gene was the second candidate revealed from the GWAS on FCR, FI, and BWG. Previous functional studies of *GTF2I*, and the corresponding chromosomal region, also revealed effects on weight- and growth-related traits. In murine fibroblasts, different isoforms of *GTF2I* were shown to be involved in the transcriptional regulation of growth factor signaling pathways^29^. Additionally, functional analyses in mice revealed that heterozygous *GTF2I* knock-outs grow slower and have reduced body weights in adult stages^30^. Furthermore, the corresponding genomic region including *GTF2I* on human chromosome 7q11.23 is affected by chromosomal rearrangements (including up to 26 genes) resulting in multiple characteristics of the Williams-Beuren syndrome^31^,^32^. Williams-Beuren syndrome is a multisystem disorder, and hemizygosity of affected genes is associated with delayed growth and glucose intolerance. No evidence for loss of heterozygosity was observed at the *GTF2I* SNP locus based on the observed allele frequency.

Both selected candidate genes, *AGK* and *GTF2I*, represent different biological aspects with influence on the complex processes involved in individual FCE. Nevertheless, an essential next step will be to validate the robustness of the association of those candidates in independent broiler populations, and to clarify the functional contribution of *AGK* and *GTF2I* to FCE in chickens.

In summary, this study provides novel insight into the genetics of FCE in a commercial meat-type chicken population selected for FCE over many generations. Although loci with large effects on FCE seem to be mostly fixed in the used population, the analyses revealed genetic factors beyond the basic knowledge of feed efficiency. Using the advantages of Bayesian GWAS and combining it with a GLM approach, a list of QTL regions for body weight and FCE traits was discovered. Finally, the analyses of coding SNP in selected candidate genes *AGK* and *GTF2I* provide evidence for association with FCE traits. A strategy to unravel the high biological complexity of FCE traits in further GWAS might imply a detailed focus on genes affecting the efficient nutrient utilization, itemized by amino acids, fat, and carbohydrates rather than summing all processes in the consideration of FCR.

## Materials and Methods

### Animals and phenotypes

Birds of the commercial broiler line A, which is under genetic selection for broiler growth traits, especially for average daily gain and feed conversion, were provided, raised, and sampled by Cobb-Vantress (Siloam Springs, AR, USA). An initial population of 5000 male birds was conventionally fed for the first five weeks of life. At 36 days of age, broilers were weighed (BW36), and a subpopulation of the 1000 heaviest birds was selected for the feed conversation testing in individual pens between days 39 to 46. Recorded phenotypic data were start and final weight (BW39 and BW46), total feed intake (FI), and total weight gain (BWG) recorded over the trial period. Feed conversion ratio (FCR) was calculated as the ratio of FI and BWG. At the end of the feed conversation test, blood samples from the branchial vein were collected in anticoagulant tubes for DNA extraction. Samples were collected from a commercial flock under the guidance of the local committees for the care and use of animals following the Cobb-Vantress Inc. Animal Welfare Policy. The experimental protocol was carried out in accordance with the approved guidelines for safeguarding good scientific practice at the institutions in the Leibniz Association.

### Genotyping and data processing

Extraction of genomic DNA was performed using Qiagen 96-well extraction kit (Qiagen, Hilden, Germany). A total of 864 DNA samples were genotyped employing chicken 60 K SNP Beadchips (Illumina, San Diego, CA, USA) and analyzed by the Illumina GenomeStudio Genotyping Module (v1.9.4). Three and two samples were excluded from analysis due to missing phenotypic data and sample call rates <0.99, respectively. In total, 44355 SNP markers were further examined after meeting the following selection criteria: (i) SNP call rate >95%, (ii) minor allele frequency ≥3%, and (iii) Chi^2^-test for deviation of Hardy-Weinberg equilibrium of p ≥ 0.0001. Missing genotypes in the filtered dataset were imputed using fastPHASE (v1.2) with 10 random starts and 50 iterations of the EM algorithm^33^. All SNP with no assigned chromosome or linkage group based on the current chicken (*Gallus gallus*) genome assembly (Galgal4) were removed and led to 44035 mapped SNP being used for association and linkage analyses. Population structure and unequal genetic distances within an analyzed population could affect the results of a GWAS and are major sources for false positive associations^34^. To evaluate whether the relationship between used individuals has to be considered for GWAS, the population stratification was tested employing the SNPRelate R package^35^. Thereby, multidimensional scaling analysis was performed using identity-by-state distances between individuals.

### Linkage analysis

Linkage disequilibrium (LD) (r^2^) between mapped SNP markers was calculated for each chromosome employing Haploview (v4.2)^36^. The output dataset was used to calculate average distances and r^2^ of adjacent markers. Additionally, the LD of markers included in non-overlapping sliding 1-kb windows were averaged leading to 30,000 windows covering marker distances from 0 to 30,000 kb with corresponding r^2^ values. LD values of the 1 kb windows were plotted against physical distances and nonlinear regression curves were generated by fitting a four-parameter Weibull function (type-1) using R software with ‘drc’ package (v2.5–12; https://cran.r-project.org/web/packages/drc/index.html). Based on the curves r^2^ values at distances of 10 kb, 100 kb, 500 kb, 1 Mb, and 10 Mb were estimated and compared between macro-, intermediate, and micro-chromosomes. The extent of LD was defined as ‘useful’ with r^2^ ≥ 0.25^37^. Markers that exceed this threshold in the pairwise linkage analysis were assumed to share evidence in association analyses and were considered as useful for QTL mapping.

### Whole-genome association analysis

#### Single-marker approach

Genome-wide association analysis using single marker information was performed for BW36, BW49, FI, BWG, and FCR using generalized linear models (GLM) implemented in JMP Genomics 6 (SAS Institute, Cary, NC, USA). The following statistical model was used:


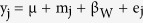


where y_j_ is the observation of the body weight and FCE traits; μ is the overall population mean for each trait; m_j_ is the fixed effect of j-th numeric genotypes; β_W_ is linear effect of BW39 as covariate, which is considered in the analyses of FI and BWG to account for differences in body weight, and e_j_ is the random residual error. Bonferroni adjustment of the genome-wide significance threshold was based on the effective number of independent tests, to account for LD between SNP markers. The number of independent tests was estimated using simpleM^38^. Therefore, the principal component parameter was set to account for 99.5% of the variance and resulted in the consideration of 19420 independent tests. Corresponding threshold p-values were set to -log_10_(p-value) = 4.3 (1/19420) and -log_10_(p-value) = 5.6 (0.05/19420) for suggestive and genome-wide significance, respectively^39^. Manhattan plots were created using the qqman R script^40^.

#### Multi-marker approach

The imputed dataset was further analyzed using the multi-marker association procedure following the Bayesian approach implemented in the web-based GenSel software (v4.73R)^41^. The Bayesian multiple regression approach has the advantage to fit all markers simultaneously. Thus, it considers the LD between markers and is independent from pedigree structure^42^. Estimations of genetic and residual variances were obtained from initial runs of the Bayes C algorithm, which is more robust to the prior genetic variance than Bayes B^43^. The proportion of SNP that were considered as having no effect on the phenotype was set to 99.5% (π = 0.995) and resulted in about 220 loci fitted in each iteration of the MCMC chain. The Bayes B algorithm was performed with 1000 iterations as burn-in, a post burn-in chain of 50000 iterations, and an output frequency of 50 iterations (1000 samples in total). The resulting model frequencies that represent the posterior probability of inclusion of a particular marker with a nonzero effect in the model were further used to calculate Bayes Factors (BF) as previously described^44^. Markers with BF that exceed the threshold of 3 – indicating increasing evidence to reject the null hypothesis – were assumed to have effects on the analyzed trait^45^.

The Bayesian GWAS was further used to analyze non-overlapping genomic windows of 1 Mb for their contribution to the genomic variance. In total 1010 1-Mb windows were obtained for the chicken genome; each window was assumed to have a theoretical proportion to the genetic variance of about 0.1% (100%/1010 windows). A window was reported as associated with the trait when it included less than 100 SNP markers and the contribution to genetic variance was at least 5 times higher than the theoretical contribution of a 1-Mb window (explained genetic variance >0.5%). Additional 1-Mb windows were developed around the highest significantly (-log_10_[p-value] ≥ 4.3) associated SNP uniquely obtained by single-marker GLM analyses.

### Candidate gene analysis

QTL regions were screened for positional candidate genes using the chicken genome resource (Ensembl chicken genome release 78, http://www.ensembl.org/Gallus_gallus/Info/Index). Single nucleotide polymorphisms located in the coding region of selected genes *GTF2I* and *AGK* were obtained from the SNP database (dbSNP, http://www.ncbi.nlm.nih.gov/SNP/). Restriction fragment length polymorphism (RFLP) assays were developed for genotyping non-synonymous SNP *GTF2I* c.2011 A > C (p.K671Q) and *AGK* c.1166 G > A (p.R389H) in a subset of 240 broilers divergent for FCR. In brief, genomic regions were amplified using primer combinations ggaAGK_f2/ggaAGK_r2 or ggaGTF2I_f1/ggaGTF2I_r1 (see Supplementary Table S1) in a standard PCR mix with SupraTherm Taq Polymerase (Genecraft, Lüdinghausen, Germany) as described elsewhere^46^. Overnight digestion of amplification products was performed using 5 U/μl *Dra*I and *Nsi*I restriction enzymes (New England Biolabs, Frankfurt, Germany) for *GTF2I* SNP c.2011 A > C and *AGK* SNP c.1166 G > A, respectively. Restriction fragments were separated on 2% agarose gel and analyzed. Association analyses were performed using SAS (MIXED procedure; SAS Institute). The applied statistical model was as described above for single-marker GWAS and included start weight as a covariate for the analyses of FI and BWG.

## Additional Information

**How to cite this article**: Reyer, H. *et al.* The genetics of feed conversion efficiency traits in a commercial broiler line. *Sci. Rep.* **5**, 16387; doi: 10.1038/srep16387 (2015).

## Supplementary Material

Supplementary Information

## Figures and Tables

**Table 1:** Extent of linkage disequilibrium (LD) between markers located on macro-, intermediate, and microchromosomes, genotyped with 60 K SNP chip in a commercial broiler population.

	LD (r^2^) of SNP markers with different distances						adjacent markers	
	1 kb	10 kb	100 kb	500 kb	1 Mb	10 Mb	average distance (bp)	average LD (r^2^)
Macro-chromosomes	0.43	0.37	0.26	0.14	0.087	0.005	28818	0.3515
Intermediate chromosomes	0.41	0.35	0.23	0.11	0.067	0.002	19939	0.3426
Micro-chromosomes	0.33	0.28	0.16	0.05	0.023	0.001	12739	0.2883

**Table 2:** Results of single- (generalized linear model) and multi- (Bayesian) marker genome-wide association analysis and identified QTL regions for body weight and feed efficiency traits in a commercial Cobb broiler line A population (n = 859).

Trait	Chromosome	1-Mb window^1^				Top SNP in 1-Mb window^2^				No. of features in window	Candidate genes
		Start (Mb)	End (Mb)	Explained genetic variance (%)	No. of significant SNP (GLM)^3^	SNP	Gga4 position	Bayes factor	−log_10_ (p-value)		
Body weight (36 days)	8	1.0	2.0	0.74	0	rs16617885	1865263	**3.65**	4.20	20	*PTPRC, NR5A2*
	12	10.0	11.0	0.99	1	rs14040564	10451780	**6.26**	4.02	16	*CHST13*
	12	12.0	13.0	5.64	8	rs13612706	12824824	**11.07**	**5.31**	3	*PTPRG*
	12	13.0	14.0	0.77	0	rs15658904	13345451	**4.43**	3.50	12	*PTPRG*
	14	5.0	6.0	0.99	1	rs14073523	5303105	**9.79**	**4.42**	29	*CACNA1H*
Body weight (46 days)	5	45.0	45.9	0.55	0	GGaluGA286907	45351053	**7.37**	4.09	14	*GLRX5*
	8	1.0	2.0	1.59	1	rs16617885	1865263	**17.09**	**5.21**	20	*PTPRC, NR5A2*
	12	13.0	14.0	0.66	0	rs15658904	13345451	**6.13**	3.42	13	*PTPRG*
	Z	19.2	20.2	–	1	rs14753816	19664346	1.02	**4.89**	9	*HTR1A*
Feed conversion ratio (39–46 days)	1	57.0	58.0	0.54	0	GGaluGA019865	57431192	**12.32**	3.29	14	*AGK*
	4	29.8	30.8	–	2	rs14445503	30302020	2.60	**5.04**	13	*HHIP*
	4	48.0	49.0	0.52	0	rs13520772	48928165	**9.12**	3.16	18	*PPP1R3B*
	6	6.0	7.0	0.95	0	rs14568465	6164912	**14.50**	3.57	5	*Sirt1*
	7	24.0	25.0	0.63	0	GGaluGA316995	24941566	**11.18**	3.04	7	*EPB41L5*
	7	25.0	26.0	0.53	0	rs16600400	25110366	**8.10**	2.95	13	*EPB41L5*
	17	7.0	8.0	0.74	1	rs14098962	7428434	**16.04**	**4.86**	27	*RXRA*
	17	8.9	9.9	–	1	GGaluGA117403	9418909	1.02	**6.57**	26	*NR5A1, NR6A1*
	19	2.0	3.0	0.87	0	rs14117856	2657299	**7.26**	2.66	9	*GTF2I*
	22	3.4	4.4	–	1	GGaluGA186837	3938945	1.00	**5.07**	7	*ADRA1A*
Weight gain (39–46 days)	17	8.9	9.9	–	1	GGaluGA117403	9418909	0.96	**5.29**	26	*GPR144, NR5A1, NR6A1*
	19	2.0	3.0	2.45	2	rs14117856	2657299	**6.68**	3.93	9	*GTF2I*
Feed intake (39–46 days)	5	5.0	6.0	0.92	1	rs16266739	5995394	**9.97**	**4.38**	15	*SPON1*
	5	6.0	7.0	1.44	0	GGaluGA273297	6074262	**7.28**	4.04	15	*SPON1*
	5	44.0	45.0	0.61	0	rs15718055	44548505	**8.55**	4.14	24	*PRIMA1*
	19	1.0	2.0	1.26	1	GGaluGA001282	1900055	**10.92**	**4.61**	7	*ENSGALG00000028350*
	19	2.0	3.0	0.71	0	GGaluGA125025	2382510	**3.90**	3.34	8	*GTF2I*
	26	0.0	1.0	-	1	rs15467593	535781	2.38	**4.50**	49	*KDM5b*

Associations exceeding the thresholds in Bayesian multi-marker (Bayes Factor >3) or linear single-marker (-log_10_[p-value] ≥ 4.3) analysis are highlighted in bold.

^1^Windows were either obtained from GenSel Software (Bayes B) if the explained genetic variance was >0.5%, or developed around highest associated single nucleotide polymorphism (SNP) in GLM analysis.

^2^Represented is the top SNP obtained from Bayesian approach (SNP with highest Bayes Factor).

^3^Number of markers reaching the significance threshold of -log_10_(p-value) ≥ 4.3 in generalized linear model (GLM) analysis.

**Table 3:** SNP-trait association of non-synonymous SNP located in *AGK* and *GTF2I*, as positional candidate genes for feed conversion ratio.

SNP	Trait (unit)	p-value*	Genotype LSM ± SEM (n)	Genotype LSM ± SEM (n)	Genotype LSM ± SEM (n)
*AGK* c.1166			GG	GA	AA
	BW36 (g)	0.002	2198.4^a^ ± 17.7 (61)	2251.3^b^ ± 13.3 (109)	2284.1^b^ ± 16.6 (70)
	BW46 (g)	0.783	3329.3 ± 39.8 (61)	3362.7 ± 29.8 (109)	3359.3 ± 37.1 (70)
	FCR (g/g)	0.008	1.71^a^ ± 0.06 (61)	1.86^ab^ ± 0.04 (109)	1.95^b^ ± 0.05 (70)
	BWG (g)	0.092	835.9 ± 32.3 (61)	797.0 ± 23.7 (109)	739.3 ± 23.0 (70)
	FI (g)	0.584	1370.8 ± 30.5 (61)	1381.9 ± 22.4 (109)	1344.4 ± 28.4 (70)
***GTF2I*** **c.2011**			**AA**	**AC**	**CC**
	BW36 (g)	0.842	2255.3 ± 18.2 (61)	2246.9 ± 12.8 (123)	2240.0 ± 19.0 (56)
	BW46 (g)	0.042	3294.9^a^ ± 39.3 (61)	3344.4^ab^ ± 27.7 (123)	3436.3^b^ ± 41.0 (56)
	FCR (g/g)	0.003	1.92^a^ ± 0.05 (61)	1.89^a^ ± 0.04 (123)	1.68^b^ ± 0.06 (56)
	BWG (g)	0.004	730.0^a^ ± 31.3 (61)	778.7^a^ ± 22.0 (123)	878.3^b^ ± 32.7 (56)
	FI (g)	0.043	1308.2^a^ ± 29.6 (61)	1377.7^ab^ ± 20.9 (123)	1412.5^b^ ± 30.9 (56)

Superscripts indicate significant associations (p < 0.05).

^*^Least square means (LSM) for genotype classes were compared by t-test and post-hoc corrected using Tukey-Kramer correction. SEM, standard error of the mean.
