# Supplementary material for: The genetics of feed conversion efficiency traits in a commercial broiler line
Source: Sci Rep. 2015 Nov 10;5:16387. doi: 10.1038/srep16387 (PMC4639841; doi:10.1038/srep16387)

Supporting Information

The genetics of feed conversion efficiency traits in a commercial broiler line

Henry Reyer[1], Rachel Hawken[2], Eduard Murani[1], Siriluck Ponsuksili[3], and Klaus Wimmers[1,*]

Table S1. Primer sequence and annealing temperature.

Primer	Sequence	Annealing temperature
ggaAGK_f2	GCTGACTAAAAAGAAATGGTGGATG	63°C
ggaAGK_r2	TTCCTCTGAACCCATACTTAGCC	63°C
ggaGTF2I_f1	CTCCTATTTGCCACCAAGACTG	60°C
ggaGTF2I_r1	TCTACAGCCCTGAAATGATACTGG	60°C

Table S2. Markers with suggestive evidence for association (-log10[p-value]≥4.3) obtained from single-marker GWAS using a generalized linear model for association with body weight at day 36 (BW36) and day 46 (BW46), and feed conversion ratio (FCR), total body weight gain (gain), and total feed intake (intake) recorded between days 39 and 46.

**Trait**	**Name**	**Chromosome**	**Gga4-position**	**Major allele**	**Minor allele**	**$-\log_{10}$ (p-value)**
BW36	**rs15652523**	12	10255073	C	T	4.344
BW36	**rs14042400**	12	12325087	T	C	4.576
BW36	**GGaluGA086367**	12	12407120	C	T	4.411
BW36	**rs13612700**	12	12813867	C	T	4.401
BW36	**rs13612706**	12	12824824	G	A	5.314
BW36	**rs14042911**	12	12834247	C	T	5.031
BW36	**GGaluGA086538**	12	12869207	T	C	4.770
BW36	**rs14042969**	12	12877322	G	A	4.461
BW36	**GGaluGA086552**	12	12889418	G	A	5.186
BW36	**rs14073523**	14	5303105	A	G	4.423
BW46	**rs16617885**	8	1865263	T	C	5.208
BW46	**rs14753816**	Z	19664346	T	C	4.893
FCR	**rs14445503**	4	30302020	A	G	5.042
FCR	**rs14445536**	4	30340403	T	C	5.027
FCR	**rs14098962**	17	7428434	A	G	4.864
FCR	**GGaluGA117403**	17	9418909	A	G	6.573
FCR	**GGaluGA186837**	22	3938945	T	C	5.069
gain	**GGaluGA117403**	17	9418909	A	G	5.289
gain	**GGaluGA125025**	19	2382510	G	A	4.430
gain	**rs15045375**	19	2619976	A	G	4.641
intake	**rs16266739**	5	5995394	G	A	4.380
intake	**GGaluGA001282**	19	1900055	T	G	4.606
intake	**rs15467593**	26	535781	G	A	4.496

Table S3. Markers with Bayes factors > 3 obtained from multi-marker GWAS (Bayes B) with body weight at day 36 (BW36) and day 46 (BW46), and feed conversion ratio (FCR), total body weight gain (gain), and total feed intake (intake) recorded between days 39 and 46.

Trait	Marker	Chr.	Gga4-Position	Major Allele	Minor Allele	PIP[1]	Window[2]	Bayes factor
BW36	**rs13828303**	1	16091637	C	A	0.0155	16	3.13
BW36	**rs13654742**	1	56597215	T	C	0.016	54	3.24
BW36	**rs13880135**	1	66169943	A	G	0.0315	64	6.47
BW36	**rs16617885**	8	1865263	T	C	0.018	678	3.65
BW36	**rs14633449**	8	1921475	G	A	0.0154	678	3.11
BW36	**rs15652523**	12	10255073	C	T	0.0179	780	3.63
BW36	**GGaluGA085403**	12	10423468	C	T	0.0205	780	4.16
BW36	**rs14040564**	12	10451780	A	G	0.0305	780	6.26
BW36	**rs14042400**	12	12325087	T	C	0.0307	782	6.30
BW36	**GGaluGA086367**	12	12407120	C	T	0.0278	782	5.69
BW36	**GGaluGA086413**	12	12499509	A	G	0.0185	782	3.75
BW36	**rs13612700**	12	12813867	C	T	0.0261	782	5.33
BW36	**rs13612706**	12	12824824	G	A	0.0527	782	11.07
BW36	**rs14042911**	12	12834247	C	T	0.0505	782	10.58
BW36	**GGaluGA086538**	12	12869207	T	C	0.0427	782	8.88
BW36	**rs14042969**	12	12877322	G	A	0.0229	782	4.66
BW36	**GGaluGA086552**	12	12889418	G	A	0.04	782	8.29
BW36	**rs15658904**	12	13345451	A	G	0.0218	783	4.43
BW36	**rs14073523**	14	5303105	A	G	0.0469	813	9.79
BW36	**rs14080146**	14	12739445	A	G	0.0227	820	4.62
BW36	**rs15012766**	14	12749054	T	C	0.018	820	3.65
BW36	**rs16190017**	23	3679079	A	G	0.0172	901	3.48
BW46	**rs13880135**	1	66169943	A	G	0.0158	64	3.19
BW46	**GGaluGA027053**	1	78681290	C	T	0.0189	76	3.83
BW46	**rs13904851**	1	91657705	T	C	0.0157	89	3.17
BW46	**rs14223132**	2	96851422	G	T	0.0186	290	3.77
BW46	**GGaluGA283165**	5	35619121	T	C	0.0169	580	3.42
BW46	**GGaluGA286907**	5	45351053	C	T	0.0357	590	7.37
BW46	**rs14542921**	5	45388412	T	C	0.0168	590	3.40
BW46	**rs16617885**	8	1865263	T	C	0.0791	678	17.09
BW46	**GGaluGA070723**	10	13342435	G	A	0.0172	743	3.48
BW46	**GGaluGA070786**	10	13456967	G	A	0.0159	743	3.22
BW46	**GGaluGA070801**	10	13480530	T	C	0.0169	743	3.42
BW46	**rs15652523**	12	10255073	C	T	0.0181	780	3.67
BW46	**GGaluGA085403**	12	10423468	C	T	0.0182	780	3.69
BW46	**GGaluGA086552**	12	12889418	G	A	0.0181	782	3.67
BW46	**rs15658904**	12	13345451	A	G	0.0299	783	6.13
BW46	**rs14043558**	12	13506835	T	C	0.0152	783	3.07
BW46	**rs14073523**	14	5303105	A	G	0.0243	813	4.96
BW46	**rs16078312**	22	2963790	T	C	0.0259	895	5.29
BW46	**GGaluGA186436**	22	3086141	G	A	0.0251	896	5.12

Trait	Marker	Chr.	Gga4-Position	Major Allele	Minor Allele	PIP[1]	Window[2]	Bayes factor
BW46	**GGaluGA186460**	22	3133145	A	G	0.0169	896	3.42
BWG	**GGaluGA273297**	5	6074262	T	C	0.0149	551	3.01
BWG	**rs15044979**	19	2209198	G	A	0.0185	863	3.75
BWG	**GGaluGA125025**	19	2382510	G	A	0.0272	863	5.56
BWG	**rs15045375**	19	2619976	A	G	0.0179	863	3.63
BWG	**rs14117786**	19	2627014	G	A	0.0194	863	3.94
BWG	**rs14117831**	19	2645176	A	G	0.0177	863	3.59
BWG	**rs14117856**	19	2657299	C	T	0.0325	863	6.68
BWG	**rs13573949**	19	2681657	A	C	0.0228	863	4.64
FCR	**rs13843754**	1	28771316	C	T	0.0304	27	6.24
FCR	**rs13855295**	1	39570794	C	T	0.0149	38	3.01
FCR	**rs13858766**	1	44290800	A	G	0.0152	43	3.07
FCR	**GGaluGA019865**	1	57431192	G	A	0.0583	55	12.32
FCR	**rs13892141**	1	81471686	G	A	0.0169	79	3.42
FCR	**rs13920554**	1	108548155	A	G	0.0224	106	4.56
FCR	**rs15380876**	1	108559844	C	T	0.0194	106	3.94
FCR	**rs13512686**	1	108696578	T	C	0.0167	106	3.38
FCR	**GGaluGA037855**	1	109817445	A	G	0.02	107	4.06
FCR	**rs15388609**	1	111844303	C	T	0.0245	109	5.00
FCR	**rs13923933**	1	112131514	C	T	0.0196	110	3.98
FCR	**GGaluGA038820**	1	112134703	A	G	0.0227	110	4.62
FCR	**rs13626043**	1	135239917	C	T	0.0215	133	4.37
FCR	**rs13962345**	1	154396280	G	A	0.0149	152	3.01
FCR	**rs14132542**	2	5917744	C	T	0.0166	199	3.36
FCR	**rs13535578**	2	5963262	C	T	0.0308	199	6.32
FCR	**GGaluGA132221**	2	6792781	G	A	0.0165	200	3.34
FCR	**rs14134641**	2	7401939	T	C	0.0343	201	7.07
FCR	**rs14135036**	2	7681591	T	C	0.027	201	5.52
FCR	**rs15918692**	2	22771909	A	G	0.0161	216	3.26
FCR	**GGaluGA138149**	2	25025768	C	T	0.0188	219	3.81
FCR	**GGaluGA138169**	2	25064445	G	T	0.0166	219	3.36
FCR	**GGaluGA138207**	2	25221880	G	A	0.0169	219	3.42
FCR	**GGaluGA139140**	2	29846425	C	T	0.0178	223	3.61
FCR	**GGaluGA148877**	2	59373341	G	A	0.0209	253	4.25
FCR	**GGaluGA148920**	2	59484679	G	A	0.0149	253	3.01
FCR	**GGaluGA168115**	2	127245945	G	A	0.0153	321	3.09
FCR	**GGaluGA168136**	2	127304730	G	A	0.0154	321	3.11
FCR	**rs14250384**	2	127310645	G	A	0.0167	321	3.38
FCR	**rs14324875**	3	21053537	C	T	0.0156	364	3.15
FCR	**GGaluGA213124**	3	25502785	C	T	0.0193	368	3.92
FCR	**GGaluGA214200**	3	28996678	G	A	0.0151	371	3.05
FCR	**rs14333535**	3	29084028	A	G	0.0173	372	3.50
FCR	**rs16246139**	3	29322224	G	A	0.0167	372	3.38
FCR	**rs16255490**	3	37896535	A	G	0.0171	380	3.46
FCR	**rs14344691**	3	40283758	C	T	0.0179	383	3.63

Trait	Marker	Chr.	Gga4-Position	Major Allele	Minor Allele	PIP[1]	Window[2]	Bayes factor
FCR	**GGaluGA252646**	4	32766956	T	C	0.0321	486	6.60
FCR	**rs13514521**	4	32865691	G	A	0.026	486	5.31
FCR	**rs15536055**	4	33116512	G	A	0.0204	487	4.14
FCR	**GGaluGA253043**	4	33522279	G	A	0.0178	487	3.61
FCR	**GGaluGA253069**	4	33565945	A	G	0.0199	487	4.04
FCR	**GGaluGA001230**	4	45826360	A	G	0.0149	499	3.01
FCR	**rs14464075**	4	46643862	A	G	0.0239	500	4.87
FCR	**GGaluGA257768**	4	47369576	T	C	0.0213	501	4.33
FCR	**GGaluGA258261**	4	48795080	G	A	0.0165	502	3.34
FCR	**rs14466944**	4	48912163	A	C	0.016	502	3.24
FCR	**rs13520772**	4	48928165	G	A	0.0438	502	9.12
FCR	**rs14470740**	4	52352644	G	A	0.0194	506	3.94
FCR	**rs14476628**	4	57689314	A	G	0.0209	511	4.25
FCR	**GGaluGA270670**	4	88683630	C	A	0.023	542	4.68
FCR	**rs14541348**	5	43969884	T	C	0.0189	588	3.83
FCR	**rs16528094**	6	2500493	C	T	0.0156	607	3.15
FCR	**rs14561672**	6	2530409	G	A	0.019	607	3.85
FCR	**rs14561695**	6	2540119	T	G	0.0158	607	3.19
FCR	**GGaluGA295929**	6	5908666	C	T	0.0182	610	3.69
FCR	**rs14568465**	6	6164912	C	T	0.0679	611	14.50
FCR	**GGaluGA296221**	6	6661372	G	A	0.0214	611	4.35
FCR	**GGaluGA296250**	6	6733290	G	A	0.0171	611	3.46
FCR	**rs16536974**	6	6785680	G	T	0.0265	611	5.42
FCR	**rs16589710**	7	15173552	C	A	0.0157	655	3.17
FCR	**rs14610843**	7	15292536	A	G	0.015	655	3.03
FCR	**GGaluGA000419**	7	21982256	G	A	0.0239	661	4.87
FCR	**GGaluGA000424**	7	21983654	C	T	0.0284	661	5.82
FCR	**GGaluGA315800**	7	22052393	G	A	0.0343	662	7.07
FCR	**GGaluGA316995**	7	24941566	A	G	0.0532	664	11.18
FCR	**GGaluGA317025**	7	24998750	G	T	0.023	664	4.68
FCR	**rs14619209**	7	25016341	G	A	0.0203	665	4.12
FCR	**rs14619217**	7	25030266	G	A	0.0279	665	5.71
FCR	**rs16600400**	7	25110366	A	G	0.0391	665	8.10
FCR	**GGaluGA320281**	7	32779005	C	T	0.0202	672	4.10
FCR	**rs14657279**	8	27139161	T	C	0.0195	704	3.96
FCR	**rs16648960**	8	27146450	C	A	0.0187	704	3.79
FCR	**rs16675418**	9	18235341	T	C	0.017	724	3.44
FCR	**GGaluGA344095**	9	22237961	C	T	0.0151	728	3.05
FCR	**rs14022631**	11	8272422	A	C	0.0202	758	4.10
FCR	**GGaluGA078569**	11	14506131	A	C	0.0162	764	3.28
FCR	**rs16015805**	14	14987952	C	T	0.0176	822	3.57
FCR	**GGaluGA105920**	14	14996584	C	T	0.0161	822	3.26
FCR	**GGaluGA105923**	14	15007723	C	T	0.0155	823	3.13
FCR	**rs15761991**	15	1045979	A	G	0.0167	825	3.38
FCR	**rs13628941**	15	2532274	G	A	0.016	826	3.24

Trait	Marker	Chr.	Gga4-Position	Major Allele	Minor Allele	PIP[1]	Window[2]	Bayes factor
FCR	**rs14099041**	17	7371507	G	A	0.0264	845	5.40
FCR	**rs14098962**	17	7428434	A	G	0.0746	845	16.04
FCR	**rs15029094**	17	7446860	T	C	0.0149	845	3.01
FCR	**GGaluGA117567**	17	9854644	A	G	0.0149	847	3.01
FCR	**GGaluGA119451**	18	3552209	T	C	0.0176	852	3.57
FCR	**rs14417110**	18	10337587	T	C	0.0162	859	3.28
FCR	**rs14116631**	19	1720443	A	G	0.0159	862	3.22
FCR	**rs15045375**	19	2619976	A	G	0.0275	863	5.63
FCR	**rs14117786**	19	2627014	G	A	0.0236	863	4.81
FCR	**rs14117831**	19	2645176	A	G	0.0204	863	4.14
FCR	**rs14117856**	19	2657299	C	T	0.0352	863	7.26
FCR	**GGaluGA125248**	19	2680108	T	C	0.0189	863	3.83
FCR	**rs13573949**	19	2681657	A	C	0.028	863	5.73
FCR	**rs14117953**	19	2712859	T	C	0.0172	863	3.48
FCR	**GGaluGA125650**	19	3308517	C	T	0.0229	864	4.66
FCR	**rs16177081**	21	822410	C	T	0.015	886	3.03
FCR	**rs16177210**	21	917642	T	C	0.0266	886	5.44
FCR	**GGaluGA182451**	21	1163370	C	A	0.0159	887	3.22
FCR	**rs14281947**	21	1256249	T	C	0.0168	887	3.40
FCR	**rs14283612**	21	3139273	C	T	0.0149	889	3.01
FCR	**GGaluGA183832**	21	3305396	G	A	0.0151	889	3.05
FCR	**GGaluGA183934**	21	3468723	G	A	0.0276	889	5.65
FCR	**rs16180691**	21	3803990	A	G	0.0229	889	4.66
FCR	**GGaluGA184102**	21	3851219	G	A	0.0172	889	3.48
FCR	**GGaluGA193563**	24	5548580	C	T	0.0162	909	3.28
FCR	**rs16200142**	26	1381085	G	A	0.0192	915	3.90
FCR	**GGaluGA195806**	26	1784218	T	C	0.0232	915	4.73
FCR	**rs14298585**	26	2212587	G	A	0.0328	916	6.75
FCR	**rs14304214**	27	4665638	T	C	0.0198	924	4.02
FCR	**GGaluGA200433**	27	4706192	A	G	0.0185	924	3.75
FCR	**rs14304172**	27	4714228	G	A	0.0178	924	3.61
FCR	**rs15247609**	28	2402314	T	C	0.0173	927	3.50
FCR	**rs15249787**	Z	5392249	T	C	0.0149	937	3.01
FCR	**rs15249836**	Z	5446694	A	G	0.0158	937	3.19
FCR	**rs14783270**	Z	7608156	G	T	0.015	939	3.03
FCR	**GGaluGA347157**	Z	7771500	A	G	0.0149	939	3.01
FCR	**GGaluGA347160**	Z	7798285	T	C	0.0162	939	3.28
FCR	**rs14758524**	Z	24441377	C	T	0.0423	956	8.79
FCR	**rs16106184**	Z	24704890	T	C	0.0297	956	6.09
FCR	**rs14777225**	Z	25846826	T	C	0.0166	957	3.36
FCR	**rs16775795**	Z	25979093	T	C	0.0151	957	3.05
FI	**rs13552233**	1	167234197	A	G	0.0182	165	3.69
FI	**GGaluGA163597**	2	110777337	G	A	0.0161	304	3.26
FI	**GGaluGA257493**	4	46339484	G	A	0.0162	500	3.28
FI	**rs16266911**	5	5916812	A	G	0.0272	550	5.56

Trait	Marker	Chr.	Gga4-Position	Major Allele	Minor Allele	PIP[1]	Window[2]	Bayes factor
FI	**rs16266739**	5	5995394	G	A	0.0477	550	9.97
FI	**rs14351333**	5	6015973	G	A	0.0276	551	5.65
FI	**rs14351269**	5	6045451	T	C	0.0239	551	4.87
FI	**GGaluGA273297**	5	6074262	T	C	0.0353	551	7.28
FI	**rs14351132**	5	6110662	G	T	0.0245	551	5.00
FI	**GGaluGA273179**	5	6350638	G	A	0.0154	551	3.11
FI	**GGaluGA283510**	5	36631050	G	A	0.0178	581	3.61
FI	**rs16502062**	5	43977881	A	G	0.0155	588	3.13
FI	**rs15718055**	5	44548505	A	C	0.0412	589	8.55
FI	**rs14542146**	5	44589371	T	C	0.0176	589	3.57
FI	**GGaluGA001282**	19	1900055	T	G	0.052	862	10.92
FI	**rs15837980**	19	1948068	T	C	0.0166	862	3.36
FI	**GGaluGA125025**	19	2382510	G	A	0.0192	863	3.90
FI	**GGaluGA197324**	26	3864602	C	T	0.0164	917	3.32
FI	**rs16203513**	26	3889383	T	C	0.016	917	3.24
FI	**rs16203593**	26	3923956	C	T	0.0214	917	4.35

[1]PIP, posterior probability of inclusion.
[2]Number of window containing the marker.

Figure S1. Multidimensional scaling analysis based on identity by state (IBS) distances between samples of a population (n=862) of commercial broiler line A chicken.

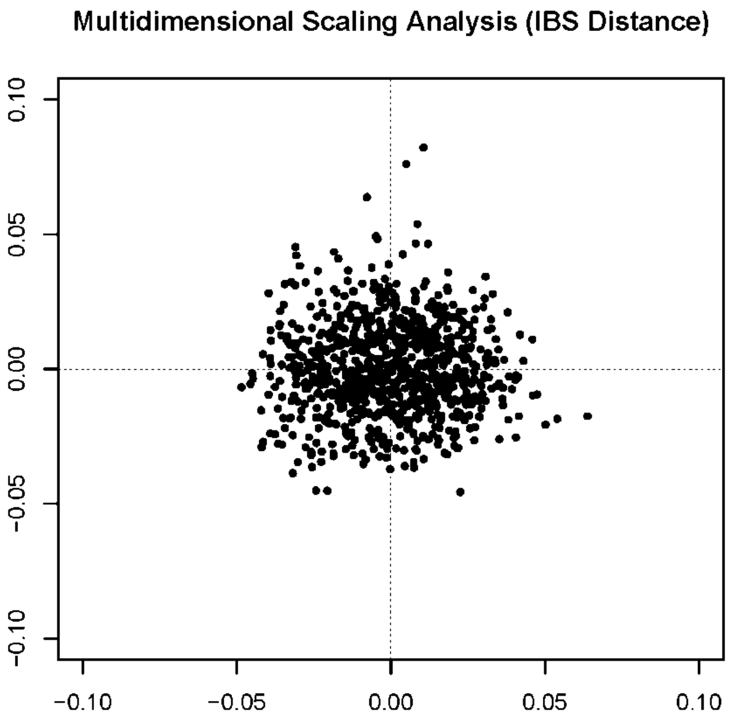

Supplement: Supplementary Information [file srep16387-s1.pdf]
